# Patatin-like phosphatase domain-containing 3 genotype and quality of dietary fat modify the liver adiposity in men

**DOI:** 10.1007/s00394-026-04031-6

**Published:** 2026-06-29

**Authors:** Milla-M. Tauriainen, Maria A. Lankinen, Juhana M. Hakumäki, Olli M. Lahtinen, Minna Husso, Jyrki J. Ågren, Markku Laakso, Ursula S. Schwab

**Affiliations:** 1 Unit of Endoscopy, Department of Medicine, Kuopio University Hospital, North Savo Wellbeing County, Finland; 2 School of Medicine, Institute of Public Health and Clinical Nutrition, University of Eastern Finland, Kuopio, Finland; 3 Diagnostic Imaging Centre, Department of Clinical Radiology, Kuopio University Hospital, North Savo Wellbeing County, Finland; 4 Institute of Clinical Medicine, Unit of Radiology, University of Eastern Finland, North Savo Wellbeing County, Finland; 5 Institute of Clinical Medicine, School of Medicine, University of Eastern Finland, Kuopio, Finland; 6 HUS Diagnostic Center, Helsinki University and Helsinki University Hospital, Helsinki, Finland; 7 Institute of Biomedicine, School of Medicine, University of Eastern Finland, Kuopio, Finland; 8 Institute of Clinical Medicine, Internal Medicine, University of Eastern Finland, Kuopio, Finland; 9 Department of Medicine, Kuopio University Hospital, North Savo Wellbeing County, Finland; 10 Department of Medicine, Endocrinology and Clinical Nutrition, Department of Medicine, Kuopio University Hospital, North Savo Wellbeing County, Finland

**Keywords:** Patatin-like phosphatase domain-containing 3 gene, Diet fat quality, Liver fat content, Liver steatosis, Liver magnetic resonance imaging, Liver magnetic resonance spectroscopy, Metabolic dysfunction-associated steatotic liver disease (MASLD)

## Abstract

**Background:**

Patatin-like phosphatase domain-containing 3 (*PNPLA3*) gene and dietary fat are important factors for metabolic dysfunction-associated steatotic liver disease (MASLD).

**Objective:**

We studied the impact of dietary fat quality modification on liver adiposity in men homozygotes for *PNPLA3* (GG, carriers of the risk allele and CC, non-carriers).

**Methods:**

Ninety-eight men (age: 67.8 ± 4.2 years, body mass index: 27.2 ± 2.5 kg/m^2^), homozygous for *PNPLA3* rs738409 variant (I148M), randomly assigned for two diet intervention arms, participated in a 12-week diet intervention. Recommended diet (RD) arm ate fat according to the National and Nordic nutrition recommendations, average diet (AD) arm ate according to the average fat intake in Finland. Liver imaging by ultrasound with 2D-shear wave elastography (2D-SWE) and magnetic resonance imaging (MRI) in combination with magnetic resonance spectroscopy (MRS) were performed.

**Results:**

MRI-based liver fat proportion decreased in the RD arm (CC: from 3.8 ± 3.2 to 3.2 ± 3.3%, GG: from 3.9 ± 3.3 to 3.5 ± 3.1%, *p time* = 0.032) and increased in the AD arm (CC: from 4.1 ± 3.6 to 4.8 ± 3.9%, GG: from 4.7 ± 3.7 to 6.7 ± 5.3%, *p time* = 0.015). MRS-quantified liver saturated fat content increased in the AD arm in both genotypes of *PNPLA3* (*p time* = 0.010). Liver triglyceride concentration did not change in either arm with the CC genotype but increased in the AD arm with the GG genotype (for time and genotype interaction *p* = 0.027).

**Conclusion:**

Diet based on fat quality recommendations could be beneficial for liver health in both *PNPLA3* genotypes CC and GG. The average diet seems to be especially harmful for liver health in carriers of the *PNPLA3* risk genotype (GG).

**Supplementary Information:**

The online version contains supplementary material available at 10.1007/s00394-026-04031-6.

## Introduction

Metabolic dysfunction associated fatty liver disease (MASLD) [1], previously known as non-alcoholic fatty liver disease (NAFLD), is a common and increasing cause of chronic liver disease worldwide [2–4]. Over one third of people worldwide have MASLD [3]. The increase of MASLD is associated with dietary habits, metabolic syndrome, obesity, sedentary lifestyle, and genetic background [4–11].

Western diet including high calorie intake and excess intake of saturated fat (SFA) are risk factors for MASLD and obesity [12, 13]. SFA, but not polyunsaturated fat (PUFA) increases intrahepatic triglycerides and obesity [10, 11, 14–16]. Mediterranean and low-fat diets have been shown MASLD resolution regardless of the *PNPLA3* genotype [17]. Only around 5% of people in Finland reach the recommended SFA intake of < 10% of total energy [18]. Liver fat evaluated by magnetic resonance imaging or magnetic resonance spectroscopy (MRI/MRS) has been reported to decrease with a PUFA enriched diet [19]. MASLD associates strongly with impaired glucose tolerance and insulin resistance [20, 21].

Genetic factors are causing MASLD in 27–39% of cases [9]. The most common genetic risk factor is a genetic variant I148M (rs738409) [22, 23] of the *PNPLA3* (Patatin-like phosphatase domain containing* 3*) gene. The prevalence of *PNPLA3* risk allele G varies in different populations between 17 and 50% [24]. In Finnish population, 6% are having MASLD-risk increasing GG-homozygotes [25].

*PNPLA3* regulates the development of adipocytes and the breakdown of fats in hepatocytes and lipocytes by lipogenesis and lipolysis [26]. Intrahepatic lipid accumulation is increased in individuals with the GG genotype, and obesity enhances this increase threefold [27, 28]. rs738409 variant (I148M) of the *PNPLA3* gene is the most common genetic variant to increase the risk of advanced MASLD. This genetic variant modifies the effect of the quality of dietary fat on liver adiposity. Fish oil supplement has been reported to decrease liver fat content by 7% in the *PNPLA3* CC + CG genotype carriers compared to 1.2% increase in carriers of the GG genotype [29].

Liver imaging is used in MASLD diagnostics [30–32]. Ultrasound shear wave elastography (SWE) is the most readily available and frequently used method to assess liver fibrosis [32]. MRI is more accurate for imaging body fat composition, and capable of semiquantitative assessment of liver fat [33], although MRS is considered as the gold standard in quantitative assessment of liver fatty acid content [15]. In a meta-analysis, the *PNPLA3* GG genotype was associated with an increased risk of liver fibrosis, independent of the severity of steatosis [34]. In addition, the increased intrahepatic liver triglyceride measured by MRI was associated with the increased hepatic insulin resistance and glucose production [35–37].

There are few studies investigating the effects of the therapeutic interventions in the patients with MASLD who are the carriers of the *PNPLA3* GG genotype [17, 38–41]. Recently, in a RCT study of 250 individuals with MASLD and known *PNPLA3* genotype, both the Mediterranean and low-fat diets have been found effective in MASLD resolution evaluated by liver ultrasound, but as confounding factor, the study participants lost weight during the study [17]. In some, but not all, dietary intervention studies, *PNPLA3* genotype has interacted with liver health. Therefore, the primary aim of this study was to examine whether the effect of dietary fat modification of liver adiposity differs in the carriers of *PNPLA3* rs738409 CC and GG genotype. As a secondary aim, we studied the association of liver adiposity to liver- and glucose metabolism-associated factors and clinical characteristics.

Our hypothesis was that the study participants would benefit from the recommended quality of dietary fat, but the participants with the *PNPLA3* rs738409 risk genotype GG would benefit more than the participants with the CC genotype.

## Methods

### Study participants

Study participants (homozygotes for *PNPLA3* rs738409 SNP, I148M variant) were recruited from the METSIM cohort [42]. The study was double-blinded, neither the individuals participating in this study, nor the study personnel knew the *PNPLA3* genotype of the participants. Inclusion criteria were: *PNPLA3* rs738409 CC or GG genotype, body mass index (BMI) < 35 kg/m^2^, total cholesterol < 8 mmol/l, low density lipoprotein (LDL) cholesterol < 5 mmol/l, plasma glucose < 7 mmol/l, plasma alanine aminotransferase (ALT) < 100 U/l and age of 60–75 years. Individuals having inflammatory diseases, active oncological disease, other liver diseases beside metabolic dysfunction associated steatotic liver disease, kidney disease, unstable thyroid disease, diabetes of any type, or mental illnesses preventing the completion of the study were excluded. Excess alcohol use (≥ 30 g daily) or smoking were also exclusion criteria.

**Fig. 1:**
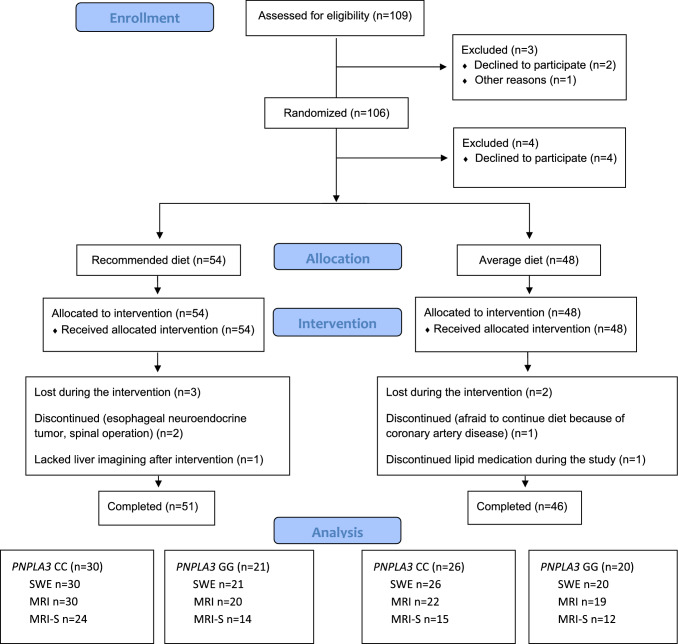
LIDIGE Study flow

One hundred and nine participants were identified as eligible for study (Fig. 1). Study participants were randomly assigned into two diet arms [recommended diet (RD) or average diet (AD)]. Altogether 102 men started the intervention [54 in the RD arm and 48 in the AD arm]. Of these participants, three discontinued the intervention. One participant discontinued lipid lowering medication during the intervention and was thereby removed from statistical analysis.

Altogether 98 men completed the intervention. Taking the *PNPLA3* genotype (CC or GG) into account there were four groups. In RD arm 31 had the CC genotype and 21 the GG genotype and in the AD arm 26 had the CC genotype and 20 the GG genotype of *PNPLA3* (Fig. 1 and Table 1).

**Table 1:** Diseases and use of medication at baseline compared within the diet arms and between the *PNPLA3* genotype groups (n = 97)

	Recommended diet		p genotype groups	Average diet		p genotype groups	*p*-value*
	CC genotype of *PNPLA3* n = 30	GG genotype of *PNPLA3* n = 21		CC genotype of *PNPLA3* n = 26	GG genotype of *PNPLA3* n = 20		
*Diseases*							
Hypertension (%)	14 (47%)	7 (33%)	0.351	7 (27%)	12 (60%)	**0.024**	0.113
Coronary artery disease (%)	5 (17%)	1 (5%)	0.476	2 (8%)	2 (10%)	0.413	0.486
Cardiac insufficiency (%)	1 (3%)	0 (0%)	0.408	0 (0%)	1 (5%)	0.259	0.566
Stroke or transient ischemic attack (%)	1 (3%)	2 (10%)	0.801	1 (4%)	0 (0%)	0.213	0.633
Cancer (%)	5 (17%)	2 (10%)	0.202	1 (4%)	0 (0%)	0.213	0.179
Rheumatoid arthritis (%)	2 (6%)	0 (0%)	0.781	1 (4%)	0 (0%)	0.213	0.411
Inflammatory bowel disease (%)	1 (3%)	1 (5%)	0.820	1 (4%)	0 (0%)	0.386	0.836
Asthma (%)	3 (10%)	3 (14%)	0.141	0 (0%)	1 (5%)	0.449	0.425
Hypothyroidism (%)	2 (7%)	0 (0%)	0.781	1 (4%)	0 (0%)	0.213	0.411
*Medications*							
Any lipid lowering medication	19 (63%)	12 (57%)	0.129	8 (31%)	9 (45%)	0.743	0.252 / 0.991
Diuretics (%)	3 (10%)	0 (0%)	0.648	3 (12%)	5 (25%)	**0.006**	0.063
Betablockers (%)	9 (29%)	3 (14%)	0.634	5 (19%)	7 (35%)	0.058	0.266
ACE / ATR (combined %)	3 / 11 (47%)	2 / 3 (24%)	0.648 / 0.081	3 / 3 (23%)	3 / 9 (60%)	0.477 / **0.010**	0.845 / **0.021**

Questionnaire-defined total (% of the group), one way ANOVA, *p < 0.05 between all study groups, bolded; antihypertensive drugs ACE, angiotensin-converting enzyme inhibitor and ATR, angiotensin receptor blocker

All except one of the 98 participants had liver imaging by 2D-SWE both at baseline and at week 12 **(**Fig. 1**)**. All 97 that had 2D-SWE, were included in the analyses. Liver MRI was technically successful for analysis of 91 out of 98 (93%) participants. MRI data could not be obtained or analyzed due to claustrophobia (n = 2), a metal fragment in the biceps (n = 1), fat–water swap artifact in the MRI (n = 3), and multicystic liver disease (n = 1). In the latter case, whole liver fat evaluation could not be performed, but a ROI-based fat evaluation and spectroscopy were analyzed (Fig. 1). MRS for lipid methyl, methylene and triglycerides were quantifiable both at the beginning and at the end of the study in 65 out of 98 (66%) participants, and MRS lipid allylic in 51 out of 98 (52%) participants (Fig. 1).

There were no differences in baseline characteristics of the participants in terms of medication or diagnosed diseases (Table 1). Alcohol consumption (analyzed both from questionnaires and food diaries) and physical exercise (questionnaire-based data) were kept constant during the study, as instructed.

The sample size calculation was based on the findings of Van Name et al. [43]. During their 12-week low n-6:n-3 PUFA ratio normocaloric diet, hepatic fat fraction decreased in *PNPLA3* rs738409 genotype group from 11 to 4% (median). Based on the reported IQR, median and n = 8, SD for hepatic fat fraction % was 5.4 in the GG genotype group at baseline. With a β = 0.20 and an α = 0.025 instead of only 0.05 to account for the fact that there were two dietary interventions and two genotypes, the sample size 11 per group was needed. Assuming 15% drop-out rate 13 participants were required per group. However, to ensure the power, MRI for 20 participants was performed per group, based on the sample sizes of Bjermo et al. [16] Rosqvist et al. [15] and Luukkonen et al. [14]. This sample size has been adequate to be able to see changes in liver fat in fat quality modification interventions without effect of genotype. However, since the effect size of the secondary outcomes is likely to be smaller than that of liver fat, we recruited 35 participants per group.

### Genotyping for PNPLA3

The genetic variant rs738409 (*PNPLA3*) was genotyped using the TaqMan SNP Genotyping Assay (Applied Biosystems, U.S.A.) according to their protocol.

### Dietary intervention and food records

Dietary intervention was advised and followed by clinical nutritionists. The participants filled four-day food records (predefined consecutive days including one weekend day) at baseline, and weeks three, seven and 11. Records were checked by a clinical nutritionist on return.

The RD arm was instructed to follow the National and Nordic nutrition recommendations regarding the fat intake [44], i.e. SFA < 10% of energy intake (E%) and unsaturated fat (UFA) > 2/3 of the total fat intake. The AD arm was advised to follow an average Finnish diet [45] with the SFA intake of 15 E% and the proportion of UFA 50% of total fat intake. Otherwise, the diet was instructed to be kept constant during the study in both study groups.

The RD arm was advised to use vegetable-oil based spread (> 60% of fat) for bread and rapeseed oil and rapeseed oil-based liquid products for cooking. Oil-based salad dressing was recommended to be used one tablespoon per day. Butter or butter-based spreads were not allowed. The use of salad dressings based on fruit juice, yogurt or sour cream were not allowed. Milk and sour milk were advised to be fat free, and yogurts low-fat (fat ≤ 1%). Low-fat cheese (≤ 17%) maximum of three to four slices per day was advised. Low-fat (< 4%) cold cuts were allowed. Fish was recommended to be eaten twice a week. Non-spiced and unsalted nuts, seeds, and almonds were allowed to be used for two tablespoons per day. All these principles are according to the Nordic and National Nutrition Recommendations [44].

The AD arm was advised to use butter-based spread for bread. Salad dressing was advised to consist of e.g. sour cream or fruit juice, not vegetable oils. For cooking it was advised to use butter and butter-based spreads. Milk and sour milk were advised to consist of at least 1.5%, and yoghurts > 1.0% fat. Cheese was advised to have more than 17% fat. Fish was allowed to be eaten at a maximum once a week. Nuts, seeds, and almonds were allowed to be used for two tablespoons per week.

To enhance compliance, the key products (spread, cooking fat and oil, and cheese) were given to the participants for free. The food records were analyzed by the AivoDiet nutrient calculation software (version 2.2.0.0; *Mashie* FoodTech Solutions Finland Oy, Turku, Finland) based on national and international analyses and international food-composition tables.

### Laboratory analyses

Concentrations of serum total, LDL and HDL cholesterol and total triglycerides, plasma glucose and insulin [46] and high-sensitivity C-reactive protein (hs-CRP) were analyzed at the University of Eastern Finland as previously described [47]. Concentrations of liver transaminases, blood count, creatinine, albumin, and bilirubin were measured at Eastern Finland Laboratory Center, ISLAB.

### Calculations for liver steatosis, liver fibrosis and diabetes associated scores

Liver steatosis, liver fibrosis, insulin sensitivity and insulin resistance scores were assessed as described in the table below.

**Table:** 

Scores	Abbreviation	Calculation
Hepatic steatosis index [48]	HSI	8 × ALT/AST + BMI(+ 2 if type 2 DM yes, + 2 if female)
NAFLD liver fat score [49]	NAFLD-LFS	− 2.89 + 1.18 × Metabolic Syndrome (Yes: 1, No: 0) + 0.45 × Type 2 Diabetes (Yes: 2, No: 0) + 0.15 × Insulin in mU/L + 0.04 × AST in U/L – 0.94 × AST/ALT
Fatty liver index [50]	FLI	(e ^0^^.953*loge (triglycerides) + 0.139*BMI + 0.718*loge (ggt) + 0.053*waist circumference − 15.745^) / (1 + e ^0.953*loge (triglycerides) + 0.139*BMI + 0.718*loge (ggt) + 0.053*waist circumference − 15.745^) * 100
Fibrosis-4 [51, 52]	FIB-4	(age × AST) / (B -Trom × √ ALT)
Aspartate aminotransferase platelet ratio index [53, 54]	APRI	(AST/40)/Trom
NAFLD fibrosis score [55]	NFS	− 1.675 + 0.037 × age (years) + 0.094 × BMI (kg/m^2^) + 1.13 × IFG/diabetes (yes = 1, no = 0) + 0.99 × AST/ALT ratio − 0.013 × Trom (× 10^9^/l) − 0.66 × Alb (g/dl)
Insulin sensitivity index [56]	MATSUDA-ISI	10,000/sqrt[(Insulin 0 min x gluc 0 min × 18) x ((Insulin 0 min + Insulin 30 min + Insulin 120 min)/3] x [(gluc 0 min + gluc 30 min + gluc 120 min) × 18/3)]
Triglyceride-Glucose Index [57, 58]	TyG	ln [fasting tg (mg/dL) x fasting gluc (mg/dL)]/2

### Liver shear wave elastography

Ultrasound (US) examinations were performed by three radiologists with five to eight years of experience in conventional US and three to five years of experience in using SWE. Logiq E9™ US-device (GE Healthcare, Chicago, IL, U.S.A) was used for all the examinations. Both gray scale imaging of the liver and the SWE were conducted with C1-6 (1–6 MHz) curved array transducer.

Participants fasted for four hours before the SWE examinations. The two-dimensional (2D) SWE was performed following the European Federation of Societies for Ultrasound in Medicine and Biology (EFSUMB) guidelines for elastography [59]. Participants laid in a supine position or under 30 degree left lateral decubitus position with the right arm elevated over their head. Measurements were made in neutral breath hold from the right liver lobe if possible. Transducer was placed between the ribs perpendicular to the surface of the skin with minimal external force. SWE measurements were performed approximately 2 cm beneath the liver capsule to avoid reverberation of artifacts placing the sampling box parallel to the liver capsule. The circular region of interest (ROI) in the sampling box was placed over the normal liver parenchyma avoiding vessels. The SWE examination was deemed reliable when ten sufficient measurements with an inter quartile range ≤ 30% of the median was obtained [59].

### Liver magnetic resonance imaging and spectroscopy

MR imaging was performed using Siemens Magnetom Aera 1.5 T scanner (Siemens, Erlangen, Germany). A body array surface coil was positioned over the liver region and MR imaging was carried out using the equipment vendor´s LiverLab application [60]. The application is a semi-automatic, user-controlled protocol that scans three sequences from the liver region. After scanning, LiverLab automatically produces calculated series of images in which e.g. are the relative fractions of fat and iron in the tissue. LiverLab segments the liver from all image frames and calculates the average fat fraction. This is shown in a report generated by LiverLab. The automatic segmentation was checked visually. If the segmentation was not successful, it was corrected manually. For quantification of liver fat, STEAM MRS spectra (TE = 12 ms) were analyzed using vendor’s Syngo.via MRS analysis software, with quantification of the lipid peak signal areas according to a Lorentzian line fit at 2.0 ppm, 1.3 ppm and 0.9 ppm. The liver triglyceride concentration (mol/L) was calculated by calibrating the line-fitted 0.9 ppm fatty acyl methyl signal with water reference signal and a liver water proton concentration of 60 mol/liter, a division by three to account for the triglyceride molecule structure with three methyl groups, corrected by the T2-relaxation rates (R2) of both liver fat and water signals.

### Statistical methods

Statistical analyses were performed with IBM SPSS Statistics for Windows, Version 27 (IBM Corp., Armonk, NY) and figure drawn with R, version 4.2.2 (R Foundation for Statistical Computing, Vienna, Austria). All tests were two-tailed and *p* values < 0.05 were considered statistically significant. The normality of the distributions of the variables was tested using a Kolmogorov–Smirnov normality test with Lilliefors significance correction. Variables with skewed distribution were transformed to base-10 logarithmic scale to achieve normal distribution. Nonparametric tests were used when a normal distribution was not achieved. One-way analysis of variance (ANOVA) with Bonferroni’s multiple comparison test was used for testing differences in baseline characteristics. Differences between the genotypes in responses to the diet, i.e. genotype-diet interaction, and changes within the diet arms (timepoints 0 vs. 12 weeks) were tested using general linear model for repeated measures. Correlation analyses were done using Spearman correlations at baseline.

### Ethical considerations and trial registration

The study protocol conforms to the ethical guidelines of the Declaration of Helsinki as reflected in a prior approval by the institution´s human research committee and has been approved by the Ethics Committee of the Northern Savo Hospital District (1408/2020). Written informed consent was obtained from each participant included in the study. The study was registered in Clinical Trials: https://ichgcp.net/clinical-trials-registry/NCT04644887.

## Results

At baseline, the study groups were otherwise similar, but the participants with the GG genotype of *PNPLA3* had lower BMI, waist circumference and fasting and two-hour glucose concentrations (Table 2, 3). According to ROI %, 23 of the study participants had over 5% fat in the liver at baseline (RD: CC n = 8/30 and GG n = 5/21, AD: CC n = 5/26 and GG n = 5/20).

**Table 2:** Clinical characteristics at baseline (n = 97)

PNPLA3 genotype	Recommended diet		Average diet		*p*-value*
	CC	GG	CC	GG	
	n = 30	n = 21	n = 26	n = 20	
Age (years)	68.8 ± 4.4	68.3 ± 4.4	66.7 ± 3.7	67.2 ± 4.2	0.666
BMI (kg/m^2^)	28.0 ± 2.5	25.5 ± 2.4	28.1 ± 2.2	26.5 ± 2.3	**4.2 × 10‾**^**4**^
Waist (cm)	102.7 ± 9.1	93.7 ± 8.4	104.2 ± 7.7	95.7 ± 7.5	**2.7 × 10⁻**^**5**^
Systolic BP (mmHg)	140 ± 20	134 ± 18	139 ± 18	139 ± 11	0.591
Diastolic BP (mmHg)	85 ± 10	85 ± 7	88 ± 10	84 ± 9	0.288
Hemoglobin (g/L)	149 ± 10	146 ± 10	153 ± 9	144 ± 10	**0.007**
Leucocyte (E9/L)	5.6 ± 2.9	5.0 ± 1.3	5.8 ± 1.1	5.4 ± 1.6	0.510
Thrombocyte (E9/L)	223 ± 36	220 ± 55	242 ± 35	237 ± 63	0.309
GGT (U/L)	33.0 ± 19.4	19.6 ± 5.8	37.9 ± 40.1	26.7 ± 9.2	0.061
ALT (U/L)	26.2 ± 12.9	23.3 ± 12.6	28.8 ± 9.4	27.8 ± 10.5	0.413
AST (U/L)	28.1 ± 7.5	26.9 ± 6.7	29.0 ± 7.1	27.0 ± 7.5	0.726
Bilirubin (umol/L)	12.6 ± 4.4	13.8 ± 7.0	12.5 ± 7.0	11.5 ± 5.8	0.684
Albumin (g/dL)	38.1 ± 3.1	38.1 ± 2.5	38.9 ± 2.2	38.7 ± 3.2	0.718
Total cholesterol (mmol/L)	4.26 ± 1.02	4.5 ± 0.79	4.64 ± 0.97	4.57 ± 1.03	0.465
HDL cholesterol (mmol/L)	1.48 ± 0.35	1.49 ± 0.36	1.32 ± 0.30	1.40 ± 0.37	0.262
LDL cholesterol (mmol/L)	2.57 ± 0.81	2.84 ± 0.76	3.00 ± 0.83	2.91 ± 0.92	0.245
Triglycerides (mmol/L)	0.98 ± 0.41	1.18 ± 0.78	1.32 ± 0.44	1.12 ± 0.49	0.143
Fasting glucose (mmol/L)	5.71 ± 0.44	5.60 ± 0.35	5.75 ± 0.33	5.83 ± 0.41	0.276
120 min glucose (mmol/L)	6.37 ± 1.54	5.85 ± 1.43	6.15 ± 1.5	5.88 ± 1.23	0.540
Fasting insulin (mU/L)	10.1 ± 6.0	7.1 ± 3.6	13.9 ± 6.7	9.1 ± 5.4	**0.001**
120 min insulin (mU/L)	72.0 ± 63.7	39.7 ± 27.1	62.3 ± 37.1	53.6 ± 46.3	0.112
Hs-CRP (mg/L)	1.13 ± 1.27	0.70 ± 0.31	1.72 ± 1.33	1.04 ± 0.87	0.119
GlycA	0.76 ± 0.06	0.76 ± 0.10	0.80 ± 0.10	0.79 ± 0.09	0.155

BMI, body mass index; BP, blood pressure; GGT, gamma-glutamyl transferase; ALT, alanine aminotransferase; AST, aspartate aminotransferase; HDL, high-density lipoprotein; LDL, low-density lipoprotein; hs-CRP, high-sensitive C-reactive protein; mean ± SD, repeated generalized linear model, *p* < 0.05 bolded, *between all the study groups at baseline, one-way ANOVA, Bonferroni

**Table 3:** Clinical characteristics at baseline and at the end of the intervention (n = 97)

Study week	Recommended diet				p time*	p time and genotype*	Average diet				p time*	p time and genotype*
	CC genotype of *PNPLA3*		GG genotype of *PNPLA3*				CC genotype of *PNPLA3*		GG genotype of *PNPLA3*			
	n = 30		n = 21				n = 26		n = 20			
	0	12	0	12			0	12	0	12		
Age (years)	68.8 ± 4.4	68.3 ± 4.4			66.7 ± 3.7	67.2 ± 4.2						
BMI (kg/m^2^)	28.0 ± 2.5	27.9 ± 2.5	25.5 ± 2.4	25.4 ± 2.5	0.054	0.893	28.1 ± 2.2	28.1 ± 2.4	26.5 ± 2.3	26.4 ± 2.3	0.414	0.636
Waist (cm)	102.7 ± 9.1	101.9 ± 9.3	93.7 ± 8.4	93.4 ± 8.8	0.051	0.631	104.2 ± 7.7	104.1 ± 8.2	95.7 ± 7.5	95.3 ± 7.3	0.447	0.753
Systolic BP (mmHg)	140 ± 20	138 ± 17	134 ± 18	129 ± 20	**0.040**	0.318	139 ± 18	139 ± 14	139 ± 11	139 ± 11	0.974	0.696
Diastolic BP (mmHg)	85 ± 10	85 ± 11	85 ± 7	83 ± 8	0.188	**0.024**	88 ± 10	90 ± 6	84 ± 9	83 ± 8	0.254	0.693
Hemoglobin (g/L)	149 ± 10	149 ± 10	146 ± 10	146 ± 9	0.437	0.817	153 ± 9	153 ± 10	144 ± 10	143 ± 10	0.734	0.871
Leucocyte (E9/L)	5.6 ± 2.9	5.7 ± 2.4	5.0 ± 1.3	5.0 ± 1.1	0.596	0.836	5.8 ± 1.1	5.8 ± 1.2	5.4 ± 1.6	5.5 ± 1.7	0.566	0.587
Thrombocyte (E9/L)	223 ± 36	211 ± 38	220 ± 55	214 ± 56	**0.002**	0.327	242 ± 35	240 ± 43	237 ± 63	241 ± 48	0.602	0.203
GGT (U/L)	33.0 ± 19.4	32.8 ± 19.2	19.6 ± 5.8	18.3 ± 5.8	0.203	0.215	37.9 ± 40.1	38.5 ± 42.3	26.7 ± 9.2	27.0 ± 12.6	0.582	0.618
ALT (U/L)	26.2 ± 12.9	27.0 ± 11.3	23.3 ± 12.6	22.1 ± 10.0	0.564	0.253	28.8 ± 9.4	29.1 ± 9.0	27.8 ± 10.5	28.2 ± 9.8	0.840	0.844
AST (U/L)	28.1 ± 7.5	28.7 ± 6.4	26.9 ± 6.7	27.7 ± 6.5	0.251	0.940	29.0 ± 7.1	29.1 ± 8.7	27.0 ± 7.5	28.2 ± 5.5	0.316	0.242
Bilirubin (umol/L)	12.6 ± 4.4	12.6 ± 4.4	13.8 ± 7.0	14.8 ± 8.8	0.943	0.736	12.5 ± 7.0	13.0 ± 7.5	11.5 ± 5.8	10.8 ± 6.2	0.640	0.290
Albumin (g/dL)	38.1 ± 3.1	38.6 ± 2.3	38.1 ± 2.5	38.5 ± 2.8	0.151	0.795	38.9 ± 2.2	38.6 ± 2.3	38.7 ± 3.2	38.7 ± 3.0	0.761	0.592
Total cholesterol (mmol/L)	4.26 ± 1.02	4.29 ± 1.05	4.5 ± 0.79	4.37 ± 0.73	0.254	0.148	4.64 ± 0.97	4.89 ± 0.93	4.57 ± 1.03	4.78 ± 1.05	**0.005**	0.730
HDL cholesterol (mmol/L)	1.48 ± 0.35	1.47 ± 0.34	1.49 ± 0.36	1.47 ± 0.35	**0.001**	0.063	1.32 ± 0.30	1.38 ± 0.30	1.40 ± 0.37	1.47 ± 0.33	**0.006**	0.776
LDL cholesterol (mmol/L)	2.57 ± 0.81	2.50 ± 0.84	2.84 ± 0.76	2.59 ± 0.71	0.463	0.058	3.00 ± 0.83	3.15 ± 0.84	2.91 ± 0.92	3.02 ± 1.02	0.082	0.857
Triglycerides (mmol/L)	0.98 ± 0.41	1.03 ± 0.43	1.18 ± 0.78	1.18 ± 0.86	0.765	0.554	1.32 ± 0.44	1.31 ± 0.35	1.12 ± 0.49	1.04 ± 0.35	0.767	0.469
Fasting glucose (mmol/L)	5.71 ± 0.44	5.72 ± 0.42	5.60 ± 0.35	5.61 ± 0.46	0.977	0.999	5.75 ± 0.33	5.80 ± 0.59	5.83 ± 0.41	5.80 ± 0.46	0.941	0.624
120 min glucose (mmol/L)	6.37 ± 1.54	5.73 ± 1.54	5.85 ± 1.43	5.82 ± 1.40	**0.033**	**0.048**	6.15 ± 1.5	6.04 ± 1.90	5.88 ± 1.23	6.18 ± 1.48	0.903	0.373
Fasting insulin (mU/L)	10.1 ± 6.0	10.4 ± 7.8	7.1 ± 3.6	6.6 ± 3.5	0.387	0.406	13.9 ± 6.7	13.2 ± 7.6	9.1 ± 5.4	10.2 ± 9.0	0.330	0.337
120 min insulin (mU/L)	72.0 ± 63.7	66.6 ± 62.9	39.7 ± 27.1	41.8 ± 27.2	0.769	0.436	62.3 ± 37.1	60.0 ± 37.3	53.6 ± 46.3	80.4 ± 95.7	0.358	0.151
Hs-CRP (mg/L)	1.13 ± 1.27	1.13 ± 1.33	0.70 ± 0.31	1.03 ± 0.93	0.382	0.387	1.72 ± 1.33	1.77 ± 1.57	1.04 ± 0.87	1.20 ± 1.07	0.813	0.975
GlycA	0.76 ± 0.06	0.77 ± 0.06	0.76 ± 0.10	0.77 ± 0.11	0.137	0.997	0.80 ± 0.10	0.79 ± 0.08	0.79 ± 0.09	0.79 ± 0.09	0.134	0.492

BMI, body mass index; BP, blood pressure; GGT, gamma-glutamyl transferase; ALT, alanine aminotransferase; AST, aspartate aminotransferase; HDL, high-density lipoprotein; LDL, low-density lipoprotein; hs-CRP, high-sensitive C-reactive protein; mean ± SD, repeated generalized linear model, *p* < 0.05 bolded, * non-parametric two independent sample test Mann–Whitney, *p* < 0.05 bolded

### Dietary intake

The study participants were compliant with the dietary instructions **(**Table 4**)**. Intake of total energy (kcal/day) or protein (E%/day) did not change during the intervention. Total fat intake (E%) decreased in the RD arm (*p time* = 0.047) and increased in the AD arm (*p time* = 2.0 × 10⁻^4^). The intake of SFA (E%) decreased in the RD arm (*p time* = 3.9 × 10⁻⁸) and increased in the AD arm (*p time* = 8.4 × 10⁻^3^) during the intervention as aimed. Additionally, the intake of both monounsaturated fat (MUFA) and PUFA increased in the RD arm (*p time* = 0.034 and *p* = 0.017, respectively), and the intake of PUFA decreased in the AD arm (*p time* = 6.6 × 10⁻⁸) (Table 4). Intake of omega-3 PUFA increased in the RD arm (*p time* = 2.0 × 10⁻⁷) and intake of both omega-6 and omega-3 PUFAs decreased in the AD arm (*p time* = 6.2 × 10^‾4^ and *p time* = 1.5 × 10^⁻5^, respectively) (Table 4). Eicosapentaenoic acid (EPA) as well as docosahexaenoic acid (DHA) intake decreased in the AD arm (*p time* = 0.006 and *p time* = 0.011, respectively). There were no differences between the genotype groups in the adherence to the diet (Table 4).

**Table 4:** Dietary intake at baseline and during the intervention (average of weeks 3, 7 and 11) (n = 97)

Study week	Recommended diet				p time	p time and genotype	Average diet				p time	p time and genotype
	CC genotype of *PNPLA3* n = 30		GG genotype of *PNPLA3* n = 21				CC genotype of *PNPLA3* n = 26		GG genotype of *PNPLA3* n = 20			
	0	interv	0	interv			0	interv	0	interv		
Energy (kcal)	2179 ± 439	2235 ± 448	2186 ± 394	2142 ± 412	0.886	0.213	2308 ± 445	2414 ± 522	2339 ± 510	2270 ± 405	0.686	0.064
Protein (E%)	16.6 ± 3.0	17.2 ± 2.3	17.5 ± 2.4	17.4 ± 1.6	0.325	0.220	16.7 ± 2.9	16.0 ± 2.3	17.0 ± 3.1	16.2 ± 2.3	**0.034**	0.636
Carbohydrate (E%)	40.6 ± 5.3	41.1 ± 5.5	42.1 ± 6.0	42.9 ± 5.4	0.266	0.748	42.2 ± 4.7	40.6 ± 5.0	43.5 ± 4.8	40.4 ± 4.7	**3.2 × 10‾**^**4**^	0.281
Fiber (g/day)	28.1 ± 12.3	29.1 ± 10.1	32.0 ± 11.4	31.1 ± 10.6	0.668	0.169	29.6 ± 8.3	28.9 ± 8.9	30.5 ± 8.4	27.4 ± 7.2	**0.024**	0.232
Fat (E%)	38.4 ± 4.4	36.7 ± 3.3	36.1 ± 5.4	35.3 ± 4.7	**0.047**	0.484	36.6 ± 4.7	38 .7 ± 4.4	35.0 ± 5.1	38.2 ± 4.7	**2.0 × 10‾**^**4**^	0.373
SFA (E%)	13.3 ± 2.6	11.0 ± 1.9	12.2 ± 2.5	10.4 ± 1.8	**3.9 × 10⁻⁸**	0.434	12.6 ± 2.0	16.3 ± 2.5	11.9 ± 2.7	15.8 ± 2.0	**8.4 × 10⁻**^**3**^**⁸**	0.427
MUFA (E%)	14.2 ± 2.4	14.8 ± 1.6	13.5 ± 2.5	14.3 ± 2.3	**0.034**	0.713	13.4 ± 2.0	12.9 ± 1.4	12.3 ± 2.2	12.8 ± 2 1	0.878	0.139
PUFA (E%)	7.3 ± 1.7	7.7 ± 1.3	7.1 ± 1.9	7.5 ± 1.3	**0.017**	0.828	7.1 ± 2.1	5.4 ± 0.7	7.4 ± 2.4	5.4 ± 0.7	**6.6 × 10⁻⁸**	0.688
Omega 6 PUFA (E%)	5.0 ± 1.1	5.2 ± 0.9	5.0 ± 1.8	5.0 ± 1.2	0.536	0.346	4.9 ± 1.5	4.2 ± 0.7	5.2 ± 2.0	4.2 ± 0.8	**6.2 × 10‾**^**4**^	0.332
Omega 3 PUFA (E%)	1.8 ± 0.6	2.1 ± 0.4	1.8 ± 0.7	2.2 ± 0.5	**2.0 × 10⁻⁷**	0.986	1.9 ± 0.6	1.4 ± 0.3	1.8 ± 0.8	1.4 ± 0.2	**1.5 × 10‾**^**5**^	0.271
EPA (E%)	0.05 ± 0.07	0.07 ± 0.04	0.07 ± 0.09	0.08 ± 0.06	0.129	0.700	0.06 ± 0.09	0.03 ± 0.03	0.07 ± 0.07	0.04 ± 0.03	**0.006**	0.989
DHA (E%)	0.14 ± 0.20	0.18 ± 0.14	0.19 ± 0.25	0.22 ± 0.16	0.178	0.913	0.17 ± 0.25	0.08 ± 0.08	0.17 ± 0.20	0.10 ± 0.08	**0.011**	0.768

E%, percent of the total energy intake; SFA, saturated fat; MUFA, monounsaturated fat; PUFA, polyunsaturated fat; EPA, eicosapentaenoic fatty acid; DHA, docosahexaenoic acid; mean ± SD, repeated generalized linear model, *p* < 0.05 bolded

The compliance was supported by the changes in the proportion of plasma CE. Plasma SFA in CE decreased in the RD arm and increased in the AD arm (*p time* = 0.011 and *p* = 2.5 × 10⁻^6^) (Supplementary Table 1). Also, in the AD arm all MUFAs increased (all *p time* < 0.001) and in both genotype groups all PUFAs decreased (all *p time* < 0.042) (Supplementary Table 1).

### Liver stiffness by ultrasound shear wave elastography

**Fig. 2:**
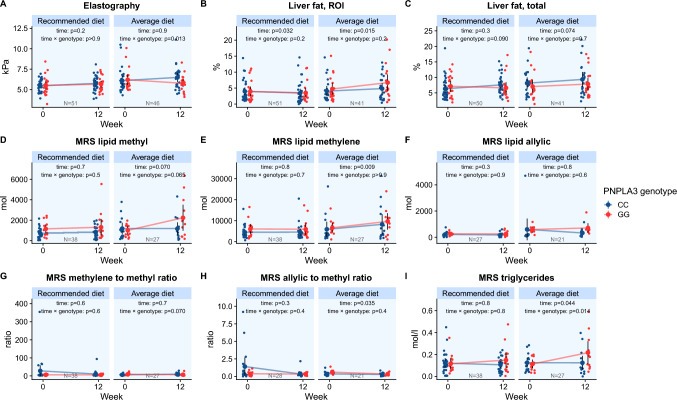
Liver elastography (**A**, n = 97), MRI-based liver fat by ROI and whole liver (**B**, n = 91 and **C**, n = 90) and MRS-based liver fat quality (**D**–**I**, n = 65) in *PNPLA3* genotypes CC and GG and the effect of dietary fat quality intervention by average diet (AD) and recommended diet (RD) (repeated generalized linear model, *P* < 0.05 bolded, 95% CI)

Liver 2D-SWE was available for all the study participants taken into analysis (n = 97, 100%) (Fig. 1). The mean values of SWE were in normal range (cut-offs of mild fibrosis to cirrhosis F1 ≥ 5.7 kPa, F2 ≥ 8.3 kPa, F3 ≥ 9.4 kPa and F4 ≥ 11.9 kPa) in all groups implying no significant liver fibrosis (Fig. 2, Supplementary Table 2). The SWE values were unchanged in the RD arm. In the AD arm, liver elasticity decreased (stiffness increased) in the participants with the *PNPLA3* CC genotype and increased (stiffness decreased) in the participants with the GG genotype (CC: from 6.1 ± 1.5 kPa to 6.5 ± 1.4 kPa and GG: from 6.2 ± 1.4 kPa to 5.8 ± 1.0 kPa, time and genotype interaction *p* = 0.020) (Fig. 2, Supplementary Table 2).

### Liver fat content by magnetic resonance imaging

MRI was available for 96 out of 98 study participants. MRI liver fat content by ROI was successful from 91 (93%) and by whole liver from 90 (92%) participants (Figs. 1 and 2, Supplementary Table 2) with a similar success rate as reported in the literature [61–63].

MRI-based liver fat (ROI, %) decreased in the RD arm (CC: from 3.82 ± 3.19% to 3.24 ± 3.32% and GG: from 3.93 ± 3.30% to 3.51 ± 3.09%, *p time* = 0.032), and increased in the AD arm (CC: from 4.05 ± 3.61% to 4.83 ± 3.90% and GG: from 4.73 ± 3.73% to 6.70 ± 5.29%, *p time* = 0.015) in both genotypes, as expected, (Fig. 2, Supplementary Table 2). The increase in liver fat was greater in the GG group after the AD, but the difference compared to the CC group did not reach statistical significance (*p* = 0.2).

### Liver fat composition evaluated by MRS

Spectroscopy of intrahepatic lipid was technically successful for 65 (66%) study participants at both time points, in which lipid methyl (-CH_3_) and methylene (-CH_2_) signals were quantified, with the allylic signal (–CH_2_–CH =) quantifiable in 51 cases of all study participants (52%). Lipid chain index (ratio of saturated methylene to methyl) could be calculated from 65 cases and an unsaturated allylic methylene to methyl index (ratio of –CH_2_–CH = to CH_3_ protons) from 51 study participants.

At baseline, liver lipid methylene, lipid allylic and triglyceride contents were lower in those with the CC than the GG genotype of *PNPLA3* (*p* = 0.023, *p* = 0.006 and *p* = 0.045). Lipid methylene signal increased with all the participants in the AD arm (CC: from 5899 ± 6534 to 8252 ± 8714 a.u. and GG: from 6621 ± 3689 to 9663 ± 6168 a.u, *p time* = 0.010 and *p* = 0.940 for time and genotype interaction) (Fig. 2, Supplementary Table 2). The liver unsaturation index (ratio of allylic to methyl signal) tended to decrease in the AD arm with the GG genotype (from 0.61 ± 0.35 to 0.33 ± 0.20 mol/L, *p* = 0.060, *p time and genotype* = 0.524) (Fig. 2, Supplementary Table 2). Similarly, liver triglyceride content did not change in the RD arm either and stayed also the same in the AD arm with the CC genotype (from 0.125 ± 0.082 to 0.125 ± 0.114 mol/L, *p* = 0.762 for time) but increased with the GG genotype (from 0.113 ± 0.064 to 0.217 ± 0.164 mol/L, *p time* = 0.082, *p time and genotype* = 0.027) (Fig. 2, Supplementary Table 2).

### Plasma lipid profile

Plasma lipid profile did not differ between the groups at baseline **(**Table 2**)**. Total cholesterol concentration increased in the AD arm in both genotypes (*p time* = 0.005). HDL cholesterol concentration decreased in the RD arm in both *PNPLA3* genotype groups (*p time* = 0.001) and increased in the AD arm in both *PNPLA3* genotype groups (*p time* = 0.006). LDL cholesterol concentration tended to increase in the AD arm in both *PNPLA3* genotypes (*p time* = 0.082) **(**Table 3**)**. The results remained unchanged after adjusting for lipid medication (data not shown).

### Plasma glucose and insulin concentrations

Fasting glucose did not change during the study (Table 3). In OGTT, the 120-min glucose decreased in both genotypes in the RD arm, but more within the subjects having CC genotype (*p* = 0.048 for time and genotype interaction). Plasma fasting or 120-min insulin did not change during the study (Table 3).

### Correlation analyses of liver imaging with liver and glucose metabolism related indices

Liver stiffness from 2D-SWE correlated negatively with insulin sensitivity index MATSUDA (*r* = − 0.209, *p* = 0.039) **(**Table 5**)**.

**Table 5:** Correlations of liver imaging with clinical characteristics, liver scores and glucose metabolism related scores at baseline

	Ultrasound	Magnetic resonance imaging		Magnetic resonance spectroscopy***					
	Shear wave elastography (kPa)	Liver fat (ROI, %)	Liver fat (whole liver, %)	MRS lipid methyl (a.u.)	MRS lipid methylene (a.u.)	MRS lipid allylic (a.u.)	Methylene to methyl ratio	Allylic to methyl ratio	MRS triglyceride concentration (mol/l)
Total, n	**97**	**91**	**90**	**77**	**77**	**61**	**72**	**62**	**77**
*Clinical characteristics*:									
BMI (kg/m^2^)	0.033	0.309**	0.392**	− 0.004	0.203	0.176	0.150	0.220	0.175
Waist	0.097	0.350**	0.397**	0.058	0.177	0.168	0.055	0.110	0.219
GGT (U/L)	0.060	0.321**	0.305**	0.059	0.248*	0.081	0.113	0.084	0.073
ALT (U/L)	0.171	0.425**	0.395**	0.159	0.320**	0.194	0.049	− 0.076	0.215
AST (U/L)	0.135	0.095	0.203	0.010	− 0.097	− 0.037	− 0.133	− 0.189	0.128
Fasting glucose (mmol/L)	0.042	0.101	− 0.136	− 0.169	0.094	0.052	0.308**	0.247	− 0.113
Total cholesterol (mmol/L)	− 0.160	0.013	− 0.088	− 0.037	0.130	0.165	0.114	0.172	− 0.005
LDL cholesterol (mmol/L)	− 0.170	0.055	− 0.056	− 0.006	0.203	0.244	0.149	0.193	− 0.007
HDL cholesterol (mmol/L)	− 0.156	− 0.351**	− 0.290**	− 0.069	− 0.194	− 0.252	− 0.120	− 0.120	− 0.094
Triglycerides (mmol/L)	0.121	0.405**	0.225*	0.207	0.337**	0.193	0.083	0.015	0.237*
Hs-CRP (mg/L)	0.072	0.219*	0.107	0.088	0.134	0.122	0.069	0.162	0.118
Glyc-A	0.078	0.386**	0.192	0.201	0.344**	0.119	0.116	0.060	0.212
*Liver scores*:									
Hepatic steatosis index (HSI)	0.103	0.477**	0.475**	0.102	0.410**	0.341**	0.170	0.195	0.206
Liver fat score (NAFLD-LFS)	0.197	0.520**	0.457**	0.168	0.366*	0.256*	0.095	0.044	0.279*
Fatty Liver Index (FLI)	0.142	0.478**	0.455**	0.100	0.303**	0.196	0.127	0.124	0.265*
AST/Platelet ratio index (APRI)	0.103	0.045	0.126	− 0.047	− 0.045	− 0.110	− 0.048	− 0.191	− 0.067
NAFLD fibrosis score (NFS)	− 0.052	− 0.171	− 0.098	− 0.121	− 0.115	− 0.111	− 0.032	0.006	− 0.056
Fibrosis-4 (FIB-4)	− 0.039	− 0.211*	− 0.092	− 0.121	− 0.199	− 0.200	− 0.073	− 0.129	− 0.025
*Glucose metabolism*:									
Matsuda index	− 0.209*	− 0.506**	− 0.433**	− 0.124	− 0.329**	− 0.242	− 0.114	− 0.093	− 0.198
HOMA-IR	0.195	0.441**	0.409**	0.111	0.284*	0.204	0.113	0.081	0.193
Triglyceride glucose index	0.114	0.405**	0.203	0.158	0.326**	0.179	0.132	0.058	0.191

BMI, body mass index; GGT, gamma-glutamyl transferase; ALT, alanine aminotransferase; AST, aspartate aminotransferase; LDL, low-density lipoprotein; HDL, high-density lipoprotein; hs-CRP, high-sensitive C-reactive protein; HOMA-IR, Homeostatic assessment of insulin resistance, Spearman, *p < 0.05 bolded, **p < 0.005, *** all SWE, MRI and MRS measurements at baseline (not only those with succeeded measurement both at the beginning and at the end of the study). Elastography in mean kPa, Liver fat in % from the region of interest (ROI) and from the whole liver

Liver fat content from MRI (both measurements from ROI and total liver %) correlated positively with waist circumference and BMI (all *p* < 0.003), with MASLD associated laboratory values gamma-glutamyl transferase (GGT), ALT and triglyceride (TG) (all *p* < 0.003), and with MASLD associated scores HSI, LFS and FLI (all *p* < 5.9 × 10‾^6^). Liver fat content correlated negatively with insulin sensitivity index Matsuda and positively with insulin resistance index HOMA-IR (all *p* < 5.7 × 10‾5). Liver fat content assessment with ROI and insulin resistance index TyG had a positive association (*p* = 6.1 × 10‾5). Liver fat correlated negatively with HDL cholesterol concentration (both *p* < 0.001). In addition, liver fat evaluated by ROI correlated negatively with liver fibrosis score FIB-4 (*p* = 0.043) and positively with inflammation parameters CRP and Glyc-A (both *p* < 0.032) (Table 5).

Liver fat composition evaluated from MRS, liver lipid methylene concentration correlated positively with MASLD-associated serum ALT, AST, triglycerides and Glyc-A, as well as HSI, LFS and FLI (all *p* < 0.007) and insulin resistance indices HOMA-IR and TyG (both *p* < 0.012) (Table 6). Liver lipid methyl concentration correlated negatively with insulin sensitivity index Matsuda (*p* = 0.003). Liver lipid allylic concentration associated positively with HIS and LFS (both *p* < 0.046). Liver SFA content (methylene to methyl concentration ratio) correlated positively with fasting glucose (*p* = 0.006), and liver triglyceride concentration correlated positively with plasma triglyceride (*p* = 0.038) and MASLD associated scores LFS and FLI (both *p* < 0.020) **(**Table 6**).**

**Table 6:** Liver and glucose metabolism related scores at baseline and at the end of the intervention (n = 97)

	Recommended diet				p time	p time and genotype	Average diet				p time	p time and genotype
	CC genotype of *PNPLA3* n = 30		GG genotype of *PNPLA3* n = 21				CC genotype of *PNPLA3* n = 26		GG genotype of *PNPLA3* n = 20			
Study week	0	12	0	12			0	12	0	12		
*Liver scores*												
Hepatic steatosis index (HSI)	35.3 ± 4.5	35.5 ± 4.4	32.4 ± 3.9	31.7 ± 3.4	0.284	0.157	36.1 ± 3.9	36.2 ± 3.9	34.7 ± 3.5	34.4 ± 3.7	0.563	0.281
Liver Fat Score (NAFLD-LFS)	− 0.43 ± 1.37	− 0.29 ± 1.54	− 1.31 ± 1.38	− 1.38 ± 1.11	0.673	0.237	0.42 ± 1.25	− 0.29 ± 1.61	− 0.41 ± 1.10	− 0.24 ± 1.54	0.534	0.222
Fatty Liver Index (FLI)	52.9 ± 22.5	52.9 ± 24.7	33.9 ± 23.0	32.2 ± 22.0	0.412	0.393	62.3 ± 19.3	62.3 ± 20.9	42.3 ± 21.6	40.3 ± 22.5	0.498	0.497
AST/platelet ratio index (APRI)	0.33 ± 0.13	0.36 ± 0.13	0.32 ± 0.11	0.34 ± 0.12	**0.016**	0.710	0.31 ± 0.08	0.31 ± 0.08	0.30 ± 0.10	0.30 ± 0.07	0.585	0.795
NAFLD Fibrosis Score (NFS)	− 0.68 ± 0.72	− 0.62 ± 0.77	− 0.77 ± 1.16	− 0.70 ± 1.02	0.396	0.999	− 1.20 ± 0.57	− 1.12 ± 0.90	− 1.31 ± 1.07	− 1.33 ± 0.89	0.535	0.769
Fibrosis-4 (FIB4)	1.83 ± 0.59	1.97 ± 0.75	1.92 ± 0.64	2.06 ± 0.68	0.057	0.990	1.56 ± 0.39	1.59 ± 0.49	1.58 ± 0.53	1.59 ± 0.45	0.726	0.754
*Glucose metabolism*												
Matsuda index	6.09 ± 4.26	6.03 ± 3.95	8.04 ± 4.42	8.41 ± 4.95	0.645	0.940	4.16 ± 2.11	4.91 ± 3.36	6.23 ± 2.99	6.37 ± 3.83	0.852	0.182
HOMA-IR	2.60 ± 1.57	2.66 ± 2.05	1.79 ± 0.98	1.68 ± 0.98	0.436	0.451	3.55 ± 1.76	3.44 ± 2.00	2.40 ± 1.50	2.72 ± 2.56	0.364	0.427
Triglyceride glucose index	4.51 ± 0.21	4.53 ± 0.20	4.55 ± 0.28	4.68 ± 0.15	0.655	0.555	4.67 ± 0.18	4.55 ± 0.29	4.58 ± 0.21	4.56 ± 0.18	0.762	0.414

HOMA-IR, Homeostatic assessment of insulin resistance, Mean ± SD, *p* < 0.05 bolded, repeated generalized linear model

While liver fat correlated positively with MASLD related clinical characteristics, liver steatosis scores and impaired glucose metabolism, the diet intervention or *PNPLA3* genotype did not change the fatty liver scores nor glucose metabolism related scores in group comparison analysis during the intervention (Table 6).

## Discussion

The novelty of our study is that it combined the effects of dietary fat modification with the effect of a genetic variant of *PNPLA3* gene (CC vs. GG). Additionally, liver fat composition was evaluated by MR-imaging and MR-spectroscopy which are rarely done in the corresponding clinical trials.

The main result in our 12-week intervention study with dietary fat modification is that liver fat decreased in the RD arm and increased in the AD arm as expected based on previous literature [64]. Liver triglyceride content evaluated with MRS increased with AD in the carriers with *PNPLA3* GG genotype. The response to dietary fat modification was affected by the *PNPLA3* genotype.

A decrease in liver fat unsaturation, as determined by the ratio of saturated methyl to allylic methyl protons from MR spectroscopy, could be detected in the participants with AD and *PNPLA3* GG genotype implying that the effect of non-optimal quality of dietary fat is reflected in the quality (unsaturation) of fat in the liver. We were also able to quantitatively assess liver triglyceride concentrations, which increased with AD in the carriers of *PNPLA3* GG genotype, also correlating with the increase observed in ROI-based MRI. Similarly, plasma lipid profile worsened in the AD arm. The measured intrahepatic triglyceride levels were similar as in a previous publication [19].

Genotyping *PNPLA3* has been suggested for MASLD patients [65, 66], but as far as a recommendation based on the genetic-based individualized treatment is lacking, genotyping is done only in selected cases. The lifestyle changes, including dietary recommendations, are still the cornerstones of the treatment of MASLD [67–69]. Accordingly with our study hypothesis, participants with AD (consuming more saturated fats) and *PNPLA3* GG genotype increased their liver total fat and saturated fat content more compared to participants with CC genotype. Our study showed that RD (consuming more unsaturated fats) is also beneficial for the carriers of *PNPLA3* CC, not only for GG genotype, and thus RD promotes liver-health to all individuals irrespective of *PNPLA3* genotype.

*PNPLA3* has been reported to facilitate the balance between hepatic triglyceride storage in mice and cells [70]. Mechanistically, the *PNPLA3* I148M variant (genotype GG) changes the lipolytic activity of *PNPLA3* on lipid droplets leading to reduced triglyceride hydrolysis and increased intrahepatic triglyceride retention. This leads to increased liver fat intake. Diets high in SFA may exacerbate hepatic fat accumulation by enhancing de novo lipogenesis and impairing lipid oxidation, whereas diets high in PUFA may promote lipid turnover and oxidation. Thus, the interaction between *PNPLA3* genotype and dietary fat quality changes are reflected in the fats accumulated in the liver tissue. In our study liver fat assessed by MR-imaging associated with obesity and ALT, GGT and TG and glucose metabolism as well as fatty liver scores as expected and supported by previous publications [68, 71, 72].

Diet interventions and *PNPLA3* genotype interaction with or without liver health have been reported previously. A recent study including 31 participants with type 2 diabetes reported in an 8-week dietary intervention that *PNPLA3* genotype modified the effect of dietary intervention [73]. In a large UK Biobank population based cohort, dietary red or processed meat intake increased liver fat content more in the carriers with *PNPLA3* GG compared to carriers with CC genotype [40]. Likely those eating more red or processed meat have a greater intake of saturated fats, like our AD arm group. Both the Mediterranean and low-fat diets have been found effective in MASLD resolution evaluated by liver ultrasound in a RCT study of 250 individuals with MASLD and known *PNPLA3* genotype, but as confounding factor, the study participants lost weight [17] unlike in our study, in which body weight remained stable.

Liver stiffness increased in the AD arm in the participants having the CC genotype and decreased significantly in the participants with the GG genotype of *PNPLA3*. The liver stiffness measured by SWE is probably of no clinical value because the study participants did not have liver fibrosis [74–76].

The strengths of our study are targeted recruitment of the participants based on their rs738409 genetic variants (I148M) of the *PNPLA3* gene and equally distributed sample sizes both in diet arms and in the genotype groups. Additionally, the participants were motivated, and therefore the number of dropouts was low. The intervention was carefully guided, and the quality of the diet was well monitored. The major confounding factors, namely body weight and physical exercise, were kept constant during the study.

Our study has also limitations. All our study participants were men because they were recruited from the METSIM cohort that consists only male [42]. Therefore, the results are not generalized to women. BMI was lower in the participants with *PNPLA3* genotype GG compared to the genotype CC, as found also in other studies [77, 78]. The analyses were therefore also corrected with BMI, but the results stayed the same. In addition, at baseline the quality of dietary fat was better than on average in Finland [79]. This could cause the effect of the RD to be smaller than the AD because participants had fat consumption closer to the RD than the AD. Also, our study participants were fairly healthy (23% of participants having MASLD), and therefore these results cannot be applied to people with MASLD only.

In addition, our study had a small sample size limiting the ability to perform subgroup and sensitivity analyses, which would have been important for assessing the robustness and consistency of the findings presented. Also, the relatively short intervention of 12 weeks limits the conclusions of long-term effects of dietary fat quality on liver fat accumulation between the *PNPLA3* GG and CC genotypes. This limitation may affect the stability and generalizability of the results.

Because 49% of the study participants had lipid lowering medication and mean age was around 68 years, our results may not be generalized in other populations and therefore should be replicated in another cohort.

## Conclusion

Dietary fat quality and *PNPLA3* genotype (CC vs GG) both affect liver fat content and liver fat composition measured by MR-imaging and MR-spectroscopy. Consuming recommended fat quality diet is beneficial for all regardless of the *PNPLA3* genotype. Consuming average Finnish fat quality diet is especially harmful for the carriers of *PNPLA3* risk genotype (GG)*.* Hence, customizing nutritional guidance concerning diet fat quality could be especially beneficial for the carriers of *PNPLA3* risk genotype.

## Supplementary Information

Below is the link to the electronic supplementary material.

Supplementary file1 (DOCX 29 KB)

## Data Availability

No datasets were generated or analysed during the current study.

## Abbreviations

AD: Average diet
ALT: Alanine aminotransferase
APRI: AST to platelet ratio index
AST: Aspartate aminotransferase
BMI: Body mass index
CE: Cholesteryl ester
CRP: C-reactive protein
E%: % Of the energy intake
FIB-4: Fibrosis-4
FLI: Fatty liver index
GGT: Gamma-glutamyl transferase
HDL-C: high-density lipoprotein cholesterol
HOMA-IR: Homeostatic assessment of insulin resistance
HSI: Hepatic steatosis index
LDL-c: Low-density lipoprotein cholesterol
LFS: Liver fat score
MASLD: Metabolic dysfunction-associated steatotic liver disease
MRI: Magnetic resonance imaging
MRS: Magnetic resonance spectroscopy
NFS: NAFLD fibrosis score
OGTT: Oral glucose tolerance test
*PNPLA3*: Patatin-like phosphatase domain-containing 3 gene
PUFA: Polyunsaturated fat
RD: Recommended diet
ROI: Region of interest
SFA: Saturated fat
SWE: Shear wave elastography
2D-SWE: 2D-shear wave elastography
TG: Triglyceride
TyG: Triglyceride glucose index
UFA: Unsaturated fat
