# Supplementary material for: Patatin-like phosphatase domain-containing 3 genotype and quality of dietary fat modify the liver adiposity in men
Source: Eur J Nutr. 2026 Jun 29;65(5):181. doi: 10.1007/s00394-026-04031-6 (PMC13314847; doi:10.1007/s00394-026-04031-6)
Supplement: Supplementary file 1 — Supplementary file1 (DOCX 29 KB) [file 394_2026_4031_MOESM1_ESM.docx]

**Supplementary table 1. Fatty acid composition of plasma cholesteryl esters at baseline and at the end of the intervention (n = 97).**

|  | **Recommended diet** | | | | **p time** | **p time and genotype** | **Average diet** | | | | **p time** | **p time and genotype** | |
| --- | --- | --- | --- | --- | --- | --- | --- | --- | --- | --- | --- | --- | --- |
|  | **CC genotype of *PNPLA3***  **n = 30** | | **GG genotype of *PNPLA3***  **n = 21** | |  |  | **CC genotype of *PNPLA3***  **n = 26** | | **GG genotype of *PNPLA3***  **n = 20** | |  |  | |
| **Study week** | **0** | **12** | **0** | **12** |  |  | **0** | **12** | **0** | **12** |  |  |  |
| **CE Mol % SFA** | 13.2 ± 0.9 | 13.1 ± 0.9 | 13.1 ± 1.3 | 12.7 ± 1.1 | **0.011** | 0.171 | 13.4 ± 1.0 | 13.9 ± 0.7 | 13.3 ± 1.0 | 13.9 ± 0.8 | **2.5 x 10⁻⁶** | 0.755 |  |
| CE Mol % 16:0 | 11.8 ± 0.6 | 11.7 ± 0.7 | 11.7 ± 1.0 | 11.4 ± 0.9 | **0.041** | 0.111 | 11.9 ± 0.8 | 12.1 ± 0.7 | 11.9 ± 0.8 | 12.1 ± 0.6 | **7.8 x 10⁻⁴** | 0.824 |  |
|  |  |  |  |  |  |  |  |  |  |  |  |  |  |
| **CE Mol % MUFA** | 24.5 ± 3.9 | 24.9 ± 3.9 | 23.4 ± 3.1 | 23.2 ± 2.2 | 0.659 | 0.346 | 23.8 ± 2.9 | 24.8 ± 2.6 | 22.7 ± 2.1 | 24.2 ± 1.8 | **1.7 x 10 ⁻⁴** | 0.506 |  |
| CE Mol % 16:1n-7 | 3.1 ± 12.0 | 3.0 ± 1.7 | 2.6 ± 1.2 | 2.3 ± 1.0 | **0.166** | 0.317 | 2.8 ± 0.8 | 3.2 ± 0.8 | 2.6 ± 0.9 | 3.1 ± 0.9 | **1.4 x 10 ⁻⁴** | 0.754 |  |
| CE Mol % 18:1n-9 | 20.1 ± 2.2 | 20.6 ± 2.3 | 19.5 ± 2.3 | 19.7 ± 1.5 | 0.183 | 0.406 | 19.7 ± 2.2 | 20.4 ± 1.9 | 18.9 ± 1.5 | 19.8 ± 1.2 | **0.001** | 0.608 |  |
|  |  |  |  |  |  |  |  |  |  |  |  |  |  |
| **CE Mol % PUFA** | 62.3 ± 4.3 | 62.0 ± 4.3 | 63.5 ± 4.1 | 64.1 ± 2.9 | 0.661 | 0.163 | 62.8 ± 3.4 | 61.3 ± 2.8 | 64.0 ± 2.8 | 2.1 ± 1.8 | **6.1 x 10⁻⁵** | 0.609 |  |
| CE Mol % 18:2n-6 | 49.9 ± 5.2 | 49.4 ± 5.4 | 52.1 ± 5.3 | 52.8 ± 4.2 | 0.959 | 0.113 | 50.4 ± 3.6 | 49.5 ± 3.3 | 51.5 ± 3.2 | 50.1 ± 2.2 | **5.2 x 10 ‾⁴** | 0.565 |  |
| CE Mol % 18:3n-3 | 0.96 ± 0.22 | 1.02 ± 0.24 | 0.97 ± 0.23 | 1.04 ± 0.23 | 0.025 | 0.618 | 0.93 ± 0.22 | 0.83 ± 0.20 | 0.99 ± 0.28 | 0.96 ± 0.22 | **0.009** | 0.162 |  |
| CE Mol % 20:5n-3 | 1.88 ± 0.76 | 1.98 ± 0.52 | 1.55 ± 0.70 | 1.71 ± 0.76 | 0.062 | 0.809 | 1.76 ± 0.68 | 1.54 ± 0.62 | 1.92 ± 0.76 | 1.79 ± 0.82 | **0.042** | 0.458 |  |
| CE Mol % 22:6n-3 | 0.91 ± 0.28 | 0.91 ± 0.26 | 0.98 ± 0.18 | 0.93 ± 0.24 | 0.322 | 0.264 | 0.97 ± 0.26 | 0.82 ± 0.23 | 1.00 ± 0.28 | 0.87 ± 0.25 | **1.2 x10 ‾⁵** | 0.772 |  |
|  |  |  |  |  |  |  |  |  |  |  |  |  |  |

CE, cholesteryl ester; mean ± SD, repeated generalized linear model, p < 0.05 bolded; SFA, saturated fatty acids; MUFA, monounsaturated fatty acids; PUFA, polyunsaturated fatty acids.

**Supplementary table 2. Liver imagining at baseline and at the end of the intervention.**

ROI, region of interest, mean ± SD, p < 0.05 bolded, repeated generalized linear model.

|  | **Recommended diet** | | | | **p time** | **p time and genotype** | **Average diet** | | | | **p time** | **p time and genotype** |
| --- | --- | --- | --- | --- | --- | --- | --- | --- | --- | --- | --- | --- |
|  | **CC genotype of *PNPLA3*** | | **GG genotype of *PNPLA3*** | |  |  | **CC genotype of *PNPLA3*** | | **GG genotype of *PNPLA3*** | |  |  |
| Study week | **0** | **12** | **0** | **12** |  |  | **0** | **12** | **0** | **12** |  |  |
|  |  |  |  |  |  |  |  |  |  |  |  |  |
| **Ultrasound (n = 97)** | **n = 30** | | **n = 21** | |  |  | **n = 26** | | **n = 20** | |  |  |
| Shear wave elastography (kPa) | 5.47 ± 0.76 | 5.76 ± 0.96 | 5.50 ± 1.17 | 5.67 ± 0.88 | 0.201 | 0.923 | 6.09 ± 1.49 | 6.49 ± 1.40 | 6.21 ± 1.36 | 5.77 ± 0.97 | 0.981 | **0.020** |
|  |  |  |  |  |  |  |  |  |  |  |  |  |
| **MRI (n = 91)** | **n = 30** | | **n = 20** | |  |  | **n = 22** | | **n = 19** | |  |  |
| Liver fat of ROI (%) | 3.82 ± 3.19 | 3.24 ± 3.32 | 3.93 ± 3.30 | 3.51 ± 3.09 | **0.032** | 0.158 | 4.05 ± 3.61 | 4.83 ± 3.90 | 4.73 ± 3.73 | 6.70 ± 5.29 | **0.015** | 0.218 |
| Liver fat whole (%) | 6.27 ± 3.49 | 7.42 ± 2.91 | 7.12 ± 3.96 | 6.62 ± 3.84 | 0.312 | 0.090 | 8.13 ± 4.20 | 9.35 ± 5.39 | 7.05 ± 4.12 | 7.84 ± 4.23 | 0.109 | 0.733 |
|  |  |  |  |  |  |  |  |  |  |  |  |  |
| **MRI spectroscopy (n = 65)** | **n = 24** | | **n = 14** | |  |  | **n = 15** | | **n = 12** | |  |  |
| Lipid methyl (a.u.) | 718 ± 540 | 842 ± 590 | 1155 ± 733 | 1318 ± 1414 | 0.676 | 0.506 | 1183 ± 1062 | 1191 ± 1080 | 1009 ± 612 | 2242 ± 2007 | 0.126 | 0.118 |
| Lipid methylene (a.u.) | 4484 ± 3151 | 4574 ± 3944 | 6056 ± 4112 | 5935 ± 4737 | 0.750 | 0.698 | 5899 ± 6534 | 8252 ± 8714 | 6621 ± 3689 | 9663 ± 6168 | **0.010** | 0.940 |
| Lipid allylic (a.u.) | 219 ± 172 | 182 ± 150 | 251 ± 128 | 422 ± 482 | 0.307 | 0.892 | 600 ± 1304 | 313 ± 298 | 592 ± 290 | 709 ± 473 | 0.879 | 0.624 |
| Methylene to methyl ratio | 27.9 ± 72.5 | 10.8 ± 18.5 | 6.0 ± 3.1 | 6.1 ± 3.1 | 0.607 | 0.622 | 6.4 ± 4.5 | 9.5 ± 7.8 | 8.9 ± 6.9 | 6.9 ± 6.9 | 0.966 | 0.129 |
| Allylic to methyl ratio | 1.34 ± 2.47 | 0.34 ± 0.45 | 0.37 ± 0.36 | 0.33 ± 0.17 | 0.258 | 0.355 | 0.36 ± 0.34 | 0.40 ± 0.47 | 0.61 ± 0.35 | 0.33 ± 0.20 | 0.060 | 0.524 |
| Triglycerides (mol/l) | 0.122 ± 0.108 | 0.107 ± 0.066 | 0.115 ± 0.083 | 0.149 ± 0.128 | 0.762 | 0.847 | 0.125 ± 0.082 | 0.125 ± 0.114 | 0.113 ± 0.064 | 0.217 ± 0.164 | 0.082 | **0.027** |

**Supplementary table 3. Liver and glucose metabolism related scores at baseline (n=97).**

|  | **Recommended diet** | | **p* genotype groups** | **Average diet** | | **p* genotype groups** | **p value**** |
| --- | --- | --- | --- | --- | --- | --- | --- |
|  | **CC genotype of *PNPLA3*** | **GG genotype of *PNPLA3*** |  | **CC genotype of *PNPLA3*** | **GG genotype of *PNPLA3*** |  |  |
|  | **n = 30** | **n = 21** |  | **n = 26** | **n = 20** |  |  |
| **Liver scores:** |  |  |  |  |  |  |  |
| Hepatic steatosis index (HSI) | 35.3 ± 4.5 | 32.4 ± 3.9 | **0.014** | 36.1 ± 3.9 | 34.7 ± 3.5 | 0.239 | **0.015** |
| Liver Fat Score (NAFLD-LFS) | -0.43 ± 1.37 | -1.31 ± 1.38 | **0.028** | 0.42 ± 1.25 | -0.41 ± -1.10 | **0.024** | **2.7 x 10‾⁴** |
| Fatty Liver Index (FLI) | 52.9 ± 22.5 | 33.9 ± 23.0 | **0.005** | 62.3 ± 19.3 | 42.3 ± 21.6 | **0.002** | **1.3 x 10‾⁴** |
| AST/platelet ratio index (APRI) | 0.33 ± 0.13 | 0.32 ± 0.11 | 0.778 | 0.31 ± 0.08 | 0.30 ± 0.10 | 0.824 | 0.758 |
| NAFLD Fibrosis Score (NFS) | -0.68 ± 0.72 | -0.77 ± 1.16 | 0.751 | -1.20 ± 0.57 | -1.31 ± 1.07 | 0.644 | **0.033** |
| Fibrosis-4 (FIB4) | 1.83 ± 0.59 | 1.92 ± 0.64 | 0.627 | 1.56 ± 0.39 | 1.58 ± 0.53 | 0.851 | 0.056 |
|  |  |  |  |  |  |  |  |
| **Glucose metabolism** |  |  |  |  |  |  |  |
| Matsuda index | 6.09 ± 4.26 | 8.04 ± 4.42 | 0.120 | 4.16 ± 2.11 | 6.23 ± 2.99 | **0.008** | **0.005** |
| HOMA-IR | 2.60 ± 1.57 | 1.79 ± 0.98 | **0.043** | 3.55 ± 1.76 | 2.40 ± 1.50 | **0.024** | **0.001** |
| Triglyceride glucose index (TyG) | 4.51 ± 0.21 | 4.55 ± 0.28 | 0.511 | 4.67 ± 0.18 | 4.58 ± 0.21 | 0.133 | 0.052 |
|  |  |  |  |  |  |  |  |

HOMA-IR, Homeostatic assessment of insulin resistance; MRI, magnetic resonance imagining; ROI, region of interest; AST, aspartate aminotransferase; mean ± SD, *between all genotypes in each diet, one-way ANOVA, **between all four study groups one-way ANOVA, p < 0.05 bolded.
